# Research on the Influence Path of Metacognitive Reading Strategies on Scientific Literacy

**DOI:** 10.3390/jintelligence11050078

**Published:** 2023-04-23

**Authors:** Yong Xie, Jingying Wang, Siqi Li, Yonghe Zheng

**Affiliations:** 1 Research Institute of Science Education, Faculty of Education, Beijing Normal University, No.19, Xinjiekouwai Str., Haidian District, Beijing 100875, China; 2 College of Education for the Future, Beijing Normal University, 18 Jinfeng Rd, Xiangzhou District, Zhuhai 519087, China

**Keywords:** scientific literacy, metacognition, reading literacy, self-efficacy, structural equation model

## Abstract

This study aims to examine influence paths of three metacognitive reading strategies (metacognitive understanding and remembering strategies, metacognitive summarizing strategies and metacognitive assessing credibility strategies) on scientific literacy, mediated by reading self-efficacy and reading literacy. The dataset included 11,420 15-year-old students from four Chinese provinces (Beijing, Shanghai, Jiangsu and Zhejiang) who took part in the Programme for International Student Assessment (PISA) in 2018. The results of structural equation model showed that metacognitive assessing credibility strategies had the greatest effect on the scientific literacy, and reading literacy played an important mediating role in the relationship between the three metacognitive reading strategies and scientific literacy. The results of the multi-group structural equation model indicated that there were significant differences in influence pathways between boys and girls, and that the reading self-efficacy of boys and girls played a different role in the impact of metacognitive summarizing strategies on scientific literacy. This study reveals the mechanism and gender difference of metacognitive reading strategies on the scientific literacy.

## 1. Introduction

The state, research organizations and educational institutions have long been interested in scientific literacy. Scientific literacy refers to an individual’s ability to engage in science-related activities, such as describing scientific phenomena, analyzing and planning scientific investigations and processing data and evidence scientifically (OECD 2019). High-level professionals with scientific literacy are necessary for the growth of the nation and society, and citizens’ participation in the discussion and decision making on socio-scientific necessitates that they possess scientific literacy. As a result, the development of scientific literacy is part of the curriculum standards in many countries. Students will play an important role in the development of science and technology and social change in the future. Attention should be paid to the development of students’ scientific literacy from the stage of basic education, which is crucial for creating a scientific and technological powerhouse.

To improve students’ scientific literacy, many researchers focus on developing students’ metacognitive reading strategies. Metacognitive reading strategies refer to students’ cognition and judgment of reading strategies (Callan et al. 2017). The students with more metacognitive reading strategies can deal with science reading tasks efficiently (Michalsky et al. 2009). Science is a semiotic subject involving the use of language, particularly in written form (Fang et al. 2008). Norris and Phillips (2003) also argued that science was in part constituted by texts. As a result, the inclusion of reading strategies in science instruction has been promoted at worldwide conferences supported by the U.S. National Foundation, in a number of well-known publications, and by academics engaging in pertinent activities. Previous studies also showed that the science lessons with reading strategies instruction contributed to students’ scientific literacy (Fang et al. 2008).

Teaching reading strategies can directly improve students’ reading literacy. Reading literacy is defined as understanding, using, evaluating, reflecting on and engaging with texts in order to achieve one’s goals, to develop one’s knowledge and potential and to participate in society (OECD 2019). It is a broad and complex concept, including an expanding set of knowledge, skills and strategies, so researchers usually evaluate reading literacy through reading ability or reading achievements. Teng (2020) examined the influence of metacognitive reading strategies teaching on learners’ reading literacy with 25 students in Grade 5 of primary school who took English as a second language as the research participants. The results showed that after the teaching intervention, the reading literacy of students in the experimental group was improved and there was a significant difference between the students in the control group and the experimental group. Additionally, both metacognitive strategies and the improvement of reading literacy can promote students’ scientific literacy. Studies have shown that students’ reading literacy and metacognitive reading strategies have positive predictive effects on their scientific literacy (O’Reilly and McNamara 2007).

The focus of this study is to examine the paths in which students’ three metacognitive reading strategies affect scientific literacy, considering reading self-efficacy and reading literacy as mediators. Previous studies have focused on the role of reading literacy and metacognitive reading strategies in scientific concept understanding and scientific reasoning, but few researchers revealed how these factors interact with each other and the effects of three sub-dimensional metacognitive reading strategies. Therefore, exploring the influence path between metacognitive reading strategies and scientific literacy is helpful to reveal the mechanism of improving students’ scientific literacy and help teachers provide effective support for students in the teaching process.

## 2. Literature Review

### 2.1. The Impact of Metacognitive Reading Strategies

Different scholars put forward different definitions of metacognition, but they all include two key features, control and knowledge, about cognitive states and processes. The former refers to the use of metacognitive strategies, while the latter includes personal knowledge, task knowledge and strategy knowledge. Flavell (1979) first proposed the concept of metacognition and defined it as the awareness of one’s cognitive processes and products, as well as the control and regulation of these mental activities and strategies. Schraw and Dennison (1994) believed that metacognition included metacognitive knowledge and metacognitive skills. Metacognitive knowledge refers to individual cognition, including declarative knowledge, procedural knowledge and conditional knowledge. Declarative knowledge is the factual knowledge and information that an individual knows, procedural knowledge is the knowledge that an individual knows how to perform certain activities, and conditional knowledge is the knowledge that an individual knows when and how to allocate resources when reusing declarative and procedural knowledge. Metacognitive skills include planning, monitoring and evaluating, which can be applied to reading-related tasks (Jacobs and Paris 1987). Planning refers to the selection of appropriate strategies and the effective allocation of resources to complete the task, monitoring refers to the observation of the progress of the task and the identification of the target for optimal performance, and evaluation refers to the assessment of the efficiency of the adjustment process and the completion of the task. According to Alexander (2008), metacognition consisted of three parts: students should make a plan before reading, monitor their understanding of the text during the process, choose appropriate strategies to deal with the text when encountering different problems and evaluate their thinking after reading. Zimmerman proposed the concept of self-regulated learning on the basis of metacognition, taking into account the regulation of behavior and motivation. Self-regulated learning refers to the process in which learners actively participate in their own learning activities from metacognitive, motivational and behavioral aspects to a certain extent (Zimmerman 1989). More and more researchers and practitioners attach importance to teaching students self-regulation learning strategies in the classroom (Schraw et al. 2006).

Brown first introduced the concept of metacognition into the field of reading, believing that the process of reading involved strategic knowledge and actions (Brown 1980). Metacognitive reading strategies refer to students’ cognition and judgment of reading strategies (Callan et al. 2017), which are used to assess the extent to which students recognize the most effective reading strategies in different reading tasks. Many researchers believe that students with more metacognitive reading strategies are more strategic and aware of reading strategies in science reading, and they are more likely to monitor their understanding and use effective reading strategies in context (Fang and Wei 2010; O’Reilly and McNamara 2007), such as reading comprehension, text summary, reasoning, etc. On the contrary, the lack of metacognitive reading strategies can explain the inability of many young learners to become effective readers. Effective readers are often thought to be strategic or constructively responsive because they efficiently allocate cognitive resources while reading (Teng 2020). However, some researchers believe that young learners do not possess metacognitive knowledge or skills, thus making metacognitive teaching ineffective (Williams and Atkins 2009). Another explanation is that the executive function of children is limited and they are unable to effectively coordinate various cognitive processes to complete tasks. Students’ learning and reflections on reading strategies will contribute to their metacognitive reading strategies, so the National Science Foundation of the United States and education researchers attach great importance to integrating reading strategies teaching into science classrooms. Fang et al. (2008) carried out an experiment on integrating reading strategies teaching into a science classroom in middle school. They worked with a school with 10 Grade 6 classes, and randomly chose six classes to act as the experimental group and four classes to act as the control group. The experimental group received one lesson per week that covered a different reading strategy, including 22 science lessons that covered reading strategies and two lessons that covered strategies summaries and reflection. However, the control group continued with the regular science teaching. The results of their practice showed that the scientific literacy of students in the science class integrated with the reading strategies teaching was more significantly improved than that in the control class.

The Programme for International Student Assessment in 2018 (PISA 2018) divides metacognitive reading strategies into three sub-dimensions: metacognitive understanding and remembering strategies, metacognitive summarizing strategies and metacognitive assessing credibility strategies (OECD 2019). The metacognitive understanding and remembering strategies measure students’ awareness and ability to use effective strategies when they are completing the task of understanding and remembering the content of the article. The metacognitive summarizing strategies measure students’ awareness and ability to use effective strategies when they are completing the task of information summarization. The metacognitive assessing credibility strategies measure students’ awareness and ability to use effective strategies to identify suspicious or uncertain information, such as sourcing and corroboration. In different text-reading situations, learners often have a variety of reading strategies to choose from. For learners skilled in different tasks, they have metacognitive reading strategies corresponding to each sub-field. They master how to choose the most suitable and efficient reading strategies to solve different tasks. Callan et al. (2017) investigated the mediating role of metacognitive reading strategies in the relationship between socioeconomic status and reading, mathematics and scientific literacy by taking the PISA 2009 data as samples, and they found that metacognitive summarizing strategies had a greater impact on scientific literacy than metacognitive understanding and remembering strategies. Metacognitive assessing credibility strategies were not evaluated until the PISA 2018, so they had not appeared in the PISA 2009.

Although metacognitive reading strategies are mainly about the cognitive ability of reading strategies, their application in other fields is limited (Salomon and Perkins 1989; Stebner et al. 2022). However, no matter which field, text is its main form of expression, which requires students to have the ability to process the text in the specific field. In particular, various phenomena and laws in science will be recorded in the form of text (Fang et al. 2008), so metacognitive reading strategies can also play an important role in the understanding of scientific concepts and argumentations. O’Reilly and McNamara (2007) tested the relationship between subject knowledge, reading skills, metacognitive reading strategies and science achievement among 1651 high school students. The results showed that students’ metacognitive reading strategies could compensate for the deficiency of subject knowledge to some extent and promote students’ science achievement. Sperling et al. (2012) investigated the relationship between metacognitive strategies knowledge and scientific achievement in 97 Grade 7 students. The results showed that students’ metacognitive strategies knowledge was an important predictor of their scientific achievement.

### 2.2. The Role of Reading Literacy and Reading Self-Efficacy

Students’ reading literacy is predicted by metacognitive reading strategies, and there is a close relationship between reading literacy and scientific literacy. Reading literacy is understanding, using, evaluating, reflecting on and engaging with texts in order to achieve one’s goals, to develop one’s knowledge and potential and to participate in society (OECD 2019). It can be assessed by reading ability because it can be viewed as an expanding set of knowledge, skills and strategies. Thus, reading literacy is related to the comprehensive application of metacognitive reading strategies. Based on the data of 3289 middle school students in the German NEPS database, Miyamoto et al. (2019) tested the mediating relationship between metacognitive reading strategies and students’ intrinsic motivation and reading comprehension ability, and the results showed that metacognitive reading strategies can significantly affect students’ reading literacy. Phakiti (2003) examined the relationship between English reading cognition and metacognitive strategies and reading achievement of 384 Thai college students, and the results showed that the use of metacognitive reading strategies was positively correlated with reading achievement, and high-reading achievers reported more metacognitive strategies than medium-reading achievers. In addition, the relationship between reading literacy and scientific literacy was also examined by researchers. Zhu (2022) analyzed the relationship between reading literacy and scientific literacy based on the PISA 2018 database, and the results showed that reading literacy was an important predictor of scientific literacy, and the effect of reading literacy on scientific literacy was far greater than that of mathematics literacy on scientific literacy. However, other studies have shown different results. Caponera et al. (2016) analyzed the impact of Italian students’ reading literacy on scientific literacy based on the TIMSS database, and the results showed that there was no apparent relationship between reading literacy and scientific literacy.

In addition to the above cognitive ability in reading, the non-cognitive ability of reading self-efficacy is also worthy of attention in science reading. Self-efficacy, proposed by American psychologist Bandura (1986), is an individual’s subjective judgment on whether he can successfully carry out a certain achievement behavior. Self-efficacy is a domain-specific concept, so it is often preceded by a specific domain and is often closely related to the competence in that domain. Reading self-efficacy refers to students’ evaluation of their reading ability. There is a one-way or reciprocal relationship between self-efficacy and ability, according to many different models, including the skill development model, the self-enhancement model, the reciprocal causality model, and so on (Chen et al. 2015). However, previous studies focused on the impact of self-efficacy on the use of reading strategies and found that students with higher self-efficacy were more willing to use reading strategies (Walker 2003). However, what this study focuses on is students’ cognition of the effectiveness of reading strategies. Metacognitive and Affective model of Self-Regulated Learning (MASRL model) showed that students’ metacognitive strategies could affect students’ self-efficacy and further affect students’ cognitive ability (Efklides 2011). This was mainly because students with metacognitive reading strategies mastered the strategies to process texts in different tasks, they would also have more confidence in their reading skills, and promoted the development of reading literacy.

### 2.3. Gender Difference in Reading and Science

The gender difference of students’ academic ability has always been an important issue in education. Although the gender gap in academic ability has narrowed or even reversed in recent years (Miller and Halpern 2014), gender differences were still found. In science subject, for example, boys’ and girls’ achievement in fairs was comparable, but boys outperformed girls at the Olympiads (Steegh et al. 2019). In reading comprehension, girls performed better than boys (Mau and Lynn 2000), and boys were more prone to dyslexia. When it came to the application of strategies, girls were better than boys (Callan et al. 2017; Slotte et al. 2001). The above differences may be due to the biological structure of male and female, but in addition to biological differences, sociocultural stereotypes were thought to reinforce these gender differences (Espinoza and Strasser 2020). Students would think that girls would definitely perform well in reading comprehension than boys (Nowicki and Lopata 2017), and girls would experience more difficulty to achieve good scientific literacy than boys. This stereotype would have different effects on the academic development of boys and girls, and also affected the path of reading related ability to scientific literacy.

## 3. Research Framework and Hypotheses

**Figure 1:**
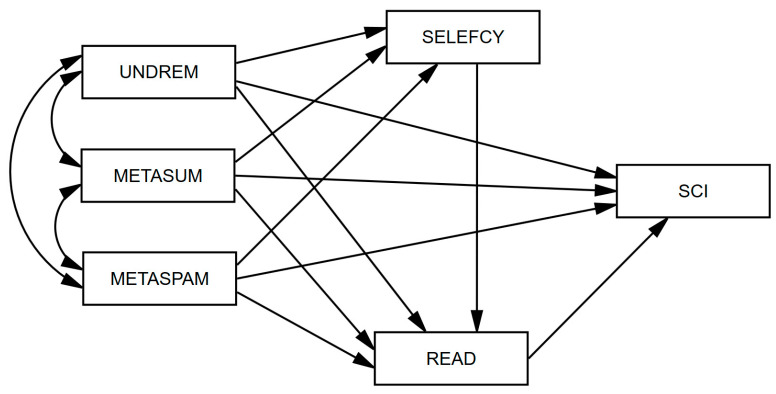
Hypothesized mediation model.

Based on the above review, we deductively assume a serial multiple mediation model (see Figure 1). The overall sequence mediating attributes of the model (three metacognitive reading strategies (UNDREM, METASUM, METASPAM) → reading self-efficacy (SELEFCY) → reading literacy (READ) → scientific literacy (SCI)). Literature on the relationship between reading literacy and metacognitive reading strategies and scientific literacy was reviewed, and it is assumed that metacognitive reading strategies will promote reading literacy and further improve scientific literacy (Miyamoto et al. 2019; Zhu 2022). Considering the domain particularity of reading self-efficacy, it is not regarded as a single mediator variable to construct the connection between reading self-efficacy and scientific literacy. Instead, based on MARSL theory, it is inferred that reading self-efficacy and reading literacy can form a pair of chain mediations (Efklides 2011), acting between the three metacognitive reading strategies and scientific literacy. In addition, due to the close correlation between the three metacognitive reading strategies, the residual covariance (represented as a double-headed arrow in Figure 1) between the three metacognitive reading strategies are freely estimated to avoid the indirect impact of estimation bias.

Based on the above model, this study aims to examine the impact of three metacognitive reading strategies (metacognitive understanding and remembering strategies (UNDREM), metacognitive summarizing strategies (METASUM) and metacognitive assessing credibility strategies (METASPAM)) on students’ scientific literacy through the mediation of reading self-efficacy and reading literacy. At the same time, based on the gender differences between boys and girls in the fields of reading and science, further exploration will be conducted on the differences in influencing mechanisms. Specifically, the following research hypotheses will be tested:

**H1.**  *Students’ metacognitive understanding and remembering strategies, metacognitive summarizing strategies and metacognitive assessing credibility strategies are positively correlated with scientific literacy.*

**H2.**  *Reading literacy plays an intermediary role between the three metacognitive reading strategies and scientific literacy.*

**H3.**  *“Reading self-efficacy → reading literacy” plays a chain-mediating role between the three metacognitive reading strategies and scientific literacy.*

**H4.**  *There are gender differences in the influence paths of three metacognitive reading strategies on scientific literacy.*

## 4. Methods

### 4.1. Participants

Based on the database provided by the Programme for International Student Assessment in 2018 (PISA 2018), this study selected the data of students from Beijing, Shanghai, Jiangsu and Zhejiang provinces of China. The project, sponsored by the Organisation for Economic Co-operation and Development (OECD), evaluates the reading, mathematics and scientific literacy of 15-year-olds around the world every three years, with a focus in 2018 on reading literacy. The PISA 2018 obtained data of 12,058 students from four provinces of China through stratified sampling, and the data of 11,420 students remained as analysis samples after the samples were screened for any missing required variables. There were 5945 boys and 5475 girls, accounting for 52.1% and 47.9% respectively. This large sample is representative of the PISA 2018 target population, thus supporting the generalizability of the results.

### 4.2. Measures

#### 4.2.1. Scientific Literacy and Reading Literacy

Literacy refers to students’ ability to solve problems by using core knowledge and skills in various fields in real life. Both scientific literacy and reading literacy were examined by situational test questions which were designed according to the assessment framework of scientific literacy and reading literacy. Based on the results of student tests, the PISA used the item response theory (IRT) to estimate the probability distribution of each student’s scientific and reading literacy (Laukaityte and Wiberg 2017), obtained 10 plausible values (PV) of scientific and reading literacy through 10 repeated estimates and assigned weight to the plausible values of each student’s achievement in literacy tests. In this study, the average of 10 plausible values was taken as students’ scientific literacy and reading literacy.

#### 4.2.2. Metacognitive Reading Strategies

Metacognitive reading strategies were evaluated using scenario-based tests. The PISA 2018 presented three scenarios, including understanding and memorizing information in text, summarizing complex two-page texts, and receiving unexpected reward information, which respectively correspond to metacognitive understanding and remembering strategies, metacognitive summarizing strategies and metacognitive assessing credibility strategies. Several strategies were given in each scenario, and students needed to evaluate the effectiveness of each strategy in solving the scenario problem. The score was from 1 to 6, with 1 indicating that the strategy is not useful at all for the reading task and 6 indicating that the strategy is very useful for the reading task (see Supplementary Material). The final score of metacognitive reading strategies in each situation was obtained by comparing the students’ evaluation with the experts’ judgment (Händel et al. 2013). The more consistent the strategy effectiveness is with the experts’ judgment, the better the student has mastered the reading strategies. Finally, the final score of each metacognitive reading strategy was converted into a mean value of OECD countries of 0 and a standard deviation of 1.

#### 4.2.3. Reading Self-Efficacy

Reading self-efficacy was assessed using a four-point Likert scale covering three items including “I am a good reader”, “I am able to understand difficult texts” and “I read fluently”. The scale is from 1 to 4, with 1 meaning strongly disagree and 4 meaning strongly agree. The scores of each question were added up to obtain the reading self-efficacy score, which was finally converted into the reading self-efficacy index with an OECD average score of 0 and a standard deviation of 1.

### 4.3. Data Analysis Strategies

SPSS 25.0 and AMOS 24.0 were used to process the data, which can be divided into three stages. Firstly, SPSS 25.0 was used to conduct descriptive statistics and correlation analysis of the data. Secondly, AMOS 24.0 was used to conduct structural equation modeling for all the sample data to verify the relationship among the three metacognitive reading strategies, reading self-efficacy, reading literacy and scientific literacy, and to test the mediating effect of reading literacy and “reading self-efficacy → reading literacy” through the bootstrap method. Finally, the multi-group structural equation model of AMOS 24.0 was used to test the differences in the influence paths between boys and girls.

## 5. Results

### 5.1. Preliminary Analysis

The mean and standard deviation of the three metacognitive reading strategies, reading self-efficacy, reading literacy and scientific literacy, as well as the Pearson correlation coefficients among variables are shown in Table 1. It should be noted that the sample included only 11,420 eligible students, which was different than the OECD average for various variables of Chinese students. Among them, the mean values of Chinese students’ metacognitive understanding and remembering strategies, metacognitive summarizing strategies, metacognitive assessing credibility strategies and reading self-efficacy were greater than 0, which all exceeded the average level of OECD countries. The mean value of metacognitive summarizing strategies was less than 0, which was lower than the average level of OECD countries.

There were differences among the three metacognitive reading strategies, reading self-efficacy, reading literacy and scientific literacy of boys and girls. Girls’ metacognitive understanding and remembering strategies, metacognitive summarizing strategies, metacognitive assessing credibility strategies and reading self-efficacy were all higher than boys and also higher than the OECD average. Boys’ metacognitive understanding and remembering strategies and metacognitive assessing credibility strategies were higher than those of OECD countries, but their metacognitive summarizing strategies were lower than those of OECD countries. Girls’ reading literacy was higher than boys’, but boys’ scientific literacy was lower than girls’.

There was a significant positive correlation between the three metacognitive reading strategies and scientific literacy, among which metacognitive assessing credibility strategies had the highest correlation with scientific literacy (r = 0.478), followed by metacognitive summarizing strategies (r = 0.378) and metacognitive understanding and remembering strategies had the lowest correlation (r = 0.334). Hypothesis 1 was verified. Except that metacognitive summarizing strategies and reading self-efficacy not having a significant correlation, metacognitive understanding and remembering strategies (r = −0.024) and metacognitive assessing credibility strategies (r = 0.061) were significantly correlated with scientific literacy. In addition, metacognitive understanding and remembering strategies (r = 0.362), metacognitive summarizing strategies (r = 0.400) and metacognitive assessing credibility strategies (r = 0.502) were significantly correlated with reading literacy, and reading literacy was strongly correlated with scientific literacy (r = 0.938).

### 5.2. Structural Equation Modeling

AMOS software was used to estimate the structural equation model constructed with three metacognitive reading strategies as independent variables, scientific literacy as a dependent variable and reading self-efficacy and reading literacy as mediating variables. The fitting results show that, with a degree of freedom of 1, the χ^2^ value was 16.01, as χ^2^/*df* = 16.01. In order to correct the influence of degrees of freedom for a chi-square test, χ^2^/*df* was usually used to evaluate the fit degree of the model, and the results were in the ideal range of 2–5. However, some researchers believe that this method is sensitive and easily affected by the sample size (Schumacker and Lomax 2016). The sample size in this study was more than 10,000, so the fitting of the structural equation model cannot be explained only by χ^2^/*df* and the chi-square test. Other main fitting indexes of the model were all within the recommended range (RMSEA = 0.036, SRMR = 0.0027, GFI = 1.000, AGFI = 0.99, NFI = 1.000, IFI = 1.000, CFI = 1.000, TLI = 0.993), indicating that the model was acceptable. It displayed good fitting degree.

**Figure 2:**
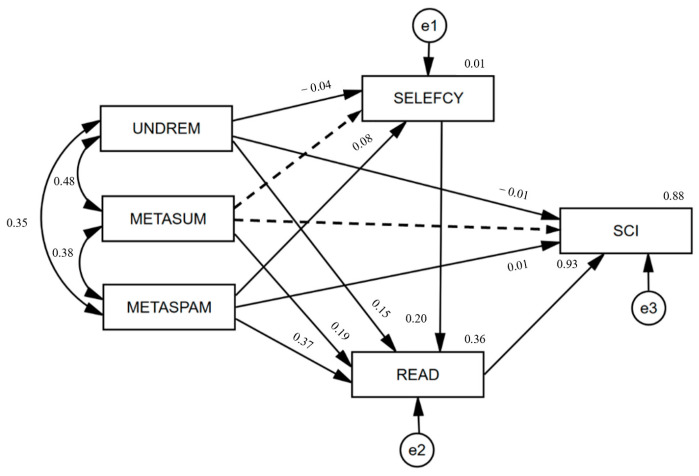
Standardized parameter estimates of the model.

Specifically, the standardized coefficients of each path in the structural equation model were shown in the Figure 2. Metacognitive understanding and remembering strategies had a significant positive predictive effect on reading literacy (β = 0.147, *p* < 0.001) and a significant negative predictive effect on reading self-efficacy (β = −0.043, *p* < 0.001) and scientific literacy (β = −0.010, *p* < 0.05). Metacognitive summarizing strategies had a positive predictive effect on reading literacy (β = 0.193, *p* < 0.001). Metacognitive assessing credibility strategies had a positive predictive effect on reading self-efficacy (β = 0.083, *p* < 0.001), reading literacy (β = 0.366, *p* < 0.001) and scientific literacy (β = 0.011, *p* < 0.01). Reading self-efficacy was a significant positive predictor of reading literacy (β = 0.196, *p* < 0.001), and reading literacy had a strong positive predictor of scientific literacy (β = 0.934, *p* < 0.001).

### 5.3. Mediation Effects

Based on the above results, the deviation-corrected percentile bootstrap method was used to further examine the mediating effects of reading self-efficacy and reading literacy on the three metacognitive reading strategies and scientific literacy. Compared with the Sobel test, the bootstrap method provided a more accurate confidence interval estimation (MacKinnon et al. 2004). Therefore, this study set bootstrap self-sampling 2000 times to estimate the mediating effect values of reading self-efficacy and reading literacy and their 95% confidence intervals. If the confidence interval does not contain 0, the mediation effect is significant.

Table 2 shows that metacognitive understanding and remembering strategy and metacognitive assessing credibility strategy had significant direct effects on scientific literacy, but with very limited effect values of −0.01 (*p* = 0.011) and 0.011 (*p* = 0.006), respectively, which should be interpreted with caution. Metacognitive summarizing strategy had no direct effects on scientific literacy. The mediating effect of reading literacy on metacognitive understanding and remembering strategy and scientific literacy was 0.137, and the bootstrap 95% confidence interval was [0.121, 0.154], which did not contain 0, indicating that reading literacy had a significant mediating effect on metacognitive understanding and remembering strategy and scientific literacy. Reading literacy also had significant mediating effects on metacognitive summarizing strategy, metacognitive assessing credibility strategy and scientific literacy, with mediating effects of 0.18 ([0.163, 0.197]) and 0.342 ([0.327, 0.356]), respectively. Hypothesis 2 was verified. “Reading self-efficacy → reading literacy” did not have a chain-mediating effect on metacognitive summarizing strategies on scientific literacy, but it did have a chain-mediating effect on metacognitive understanding and remembering strategies and metacognitive assessing credibility strategies on scientific literacy, with mediating effects of −0.008 ([−0.012, −0.004]) and 0.015 ([0.011, 0.019]), respectively, partially verifying Hypothesis 3.

In conclusion, reading literacy played an important role in the relationship between metacognitive reading strategies and scientific literacy. The relationship between metacognitive summarizing strategies and scientific literacy was completely mediated by the related paths of reading literacy, and reading literacy presented a “masking effect” in the relationship between metacognitive understanding and remembering strategies and scientific literacy. A “masking effect” was common in mediation models, which generally meant that the direct effect and indirect effect between two variables have opposite signs, leading to the total effect being masked. The “masking effect” meant that there were still mediating variables with large effects between metacognitive understanding and remembering strategies and scientific literacy that had not been included in the research field, which could be further explored in the future.

### 5.4. Multi-Group Structural Equation Model

The multi-group structural equation model was used to analyze whether there were differences in the influence paths of metacognitive reading strategies on scientific literacy among different gender groups. First, the fit degree of the unconstrained model (benchmark model) was tested, and the results showed that the fit degree was very good, RMSEA = 0.027, SRMR = 0.0017, GFI = 0.999, AGFI = 0.989, NFI = 0.999, IFI = 1.000, CFI = 1.000, TLI = 0.993, indicating that the model was acceptable for different gender groups. The model fit degree of different gender groups was further tested when all structural path coefficients limiting the influence of the three metacognitive reading strategies on scientific literacy were equal (see Table 3). The fit degree of the model was good and acceptable. On this basis, the chi-square value difference between the parallel model and the baseline model of different gender groups was compared. The results showed that the chi-square value difference between the two models reached a significant level (Δ*χ*^2^ = 38.523, Δ*df* = 11, *p* = 0.000), indicating that there were significant differences between boys and girls in the influence paths of the three metacognitive reading strategies on scientific literacy. This verified Hypothesis 4.

**Figure 3:**
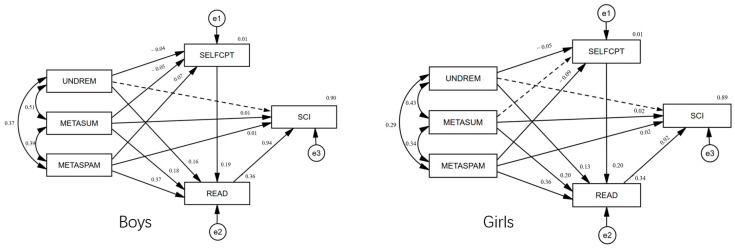
Comparison of structural equation models for boys and girls.

Figure 3 presents the results of the path structure equation model analysis of the influence of metacognitive strategies on scientific literacy in group reading for boys (*n* = 5945) and girls (*n* = 5475) based on validation model construction. Among boys and girls, metacognitive understanding and remembering strategies had no direct effect on scientific literacy, while metacognitive summarizing strategies and metacognitive assessing credibility strategies had direct effects on scientific literacy, and all three metacognitive reading strategies could indirectly affect scientific literacy through reading literacy. Among boys, three metacognitive reading strategies could affect scientific literacy through the chain mediation of “reading self-efficacy → reading literacy”, and there was a masking effect in the influence path of metacognitive summarizing strategies on scientific literacy. In girls, only metacognitive understanding and remembering strategies and metacognitive assessing credibility strategies could affect scientific literacy through the chain mediation of “reading self-efficacy → reading literacy”.

## 6. Discussion

### 6.1. The Metacognitive Assessing Credibility Strategies Had the Greatest Effect on Scientific Literacy

The Pearson correlation showed that there was a significant positive correlation between metacognitive understanding and remembering strategies, metacognitive summarizing strategies, metacognitive assessing credibility strategies and scientific literacy. It indicates that students with higher metacognitive reading strategies had higher scientific literacy. Metacognitive assessing credibility was strongly correlated with scientific literacy, metacognitive summarizing strategies and metacognitive understanding, and remembering strategies were strongly correlated with scientific literacy. Structural equation model verification further found that the total effect of the three metacognitive reading strategies on scientific literacy was positive, and the order of effect values was metacognitive assessing credibility strategies > metacognitive summarizing strategies > metacognitive understanding and remembering strategies, which indicates that the three metacognitive reading strategies could promote students’ scientific literacy. This was consistent with previous findings that metacognitive reading strategies had a positive impact on scientific literacy (O’Reilly and McNamara 2007) and metacognitive summarizing strategies had a greater impact on scientific literacy than metacognitive understanding and remembering strategies (Callan et al. 2017).

Except for metacognitive understanding and remembering strategies and metacognitive summarizing strategies, the PISA 2018 investigated metacognitive assessing credibility strategies additively. The results of this study showed that among the three metacognitive reading strategies, metacognitive assessing credibility strategies had the greatest effect on promoting scientific literacy. Among the three metacognitive strategies, metacognitive assessing credibility strategies are an important indicator of the strength of scientific literacy, which should be closely related to the ability examined by scientific literacy. Individuals with scientific literacy had strong screening ability in the face of explosive information, and they could select credible and effective evidence for demonstration (DeBoer 2000). In addition, the development of students’ scientific literacy also requires students’ ability to understand scientific text materials and to summarize information, which can be shown in the application level of metacognitive understanding and remembering strategies and metacognitive summarizing strategies, respectively. Therefore, they also would promote scientific literacy. Furthermore, since the PISA 2018 examined scientific literacy through contextualized test questions, which involved reading a large number of scientific texts (OECD 2019), the three metacognitive reading strategies of students would have a more significant role in promoting scientific literacy.

### 6.2. Reading Literacy Was Crucial in the Relationship between Metacognitive Reading Strategies and Scientific Literacy

Based on previous studies, this study further found that reading literacy was not only affected by metacognitive reading strategies (Miyamoto et al. 2019), but it also played a positive role in the relationship between metacognitive reading strategies and scientific literacy as an intermediary variable. The results of the mediating effect test showed that the total effect of the three metacognitive reading strategies on scientific literacy was positive. However, from the perspective of direct effects, only metacognitive assessing credibility strategies had a very small positive direct effect on scientific literacy, whereas metacognitive understanding and remembering strategies and metacognitive summarizing strategies had a very small negative direct effect and no direct effect on scientific literacy, respectively. However, the total indirect effects of reading literacy had a high effect size in the relationship between metacognitive reading strategies and scientific literacy, including the mediating effect path of reading literacy and the chain-mediating effect path of “reading self-efficacy → reading literacy”. Moreover, the relationship between metacognitive summarizing strategies and scientific literacy was completely mediated by the mediating path of reading literacy and the “reading self-efficacy → reading literacy” chain-mediating path. In addition, reading literacy also presented a masking effect in the relationship between comprehension and memory metacognitive strategies and scientific literacy, covering the negative of the direct effect and making the total effect positive.

Reading literacy focuses on the individual’s ability to understand, use, evaluate and reflect on text (OECD 2019), while metacognitive understanding and remembering strategies, metacognitive summarizing strategies and metacognitive assessing credibility strategies can reflect several abilities involved in reading literacy; that is, the level of metacognitive reading strategies can be used to predict the level of reading literacy (O’Reilly and McNamara 2007). The reading strategies involved in metacognitive understanding and remembering strategies and metacognitive summarizing strategies were relatively simple along with basic text-processing strategies, so the impact on scientific literacy was often limited, which was consistent with the previous results (Callan et al. 2017). Reading literacy is a comprehensive ability, which has been proven to have a great influence on scientific literacy (Zhu 2022), so it will show complete mediating or masking effects in some paths. In particular, in this study, the masking effect referred to the situation where the direct effect between metacognitive understanding and remembering strategies and scientific literacy was negative while the indirect effect was positive. This result was different from a previous study, which showed that metacognitive understanding and remembering strategies had a positive effect on scientific literacy (Callan et al. 2017). However, under the mediating effect of reading literacy in this study, the direct effect was negative, the indirect effect was positive and the total effect was positive. This may be because metacognitive understanding and remembering strategies had a limited impact on reading literacy (Callan et al. 2017), while reading literacy has a greater impact on scientific literacy (Zhu 2022), covering up the positive role that the metacognitive understanding and remembering strategy should play in scientific literacy.

### 6.3. Gender Differences Happened in the Mediating Effect of Reading Self-Efficacy

This study found results consistent with previous studies, as boys performed better than girls in scientific literacy (Steegh et al. 2019) and girls performed better than boys in reading literacy and the application of metacognitive strategies (Callan et al. 2017; Mau and Lynn 2000). The results of structural equation model difference tests of different gender groups showed that there were significant differences in the influence paths of metacognitive reading strategies on scientific literacy between boys and girls. Further analysis found a more specific conclusion that the reading self-efficacy of students of different genders was different in the relationship between metacognitive summarizing strategies and scientific literacy. The chain-mediating effect of “reading self-efficacy → reading literacy” in boys’ metacognitive summarizing strategies on scientific literacy presented a masking effect, while the chain-mediating effect of “reading self-efficacy → reading literacy” in girls’ metacognitive summarizing strategies on scientific literacy was absent.

This study found a gender difference in the influence mechanism of metacognitive reading strategies on scientific literacy between boys and girls (Nowicki and Lopata 2017). This difference is largely caused by stereotypes in social culture. Reading and text processing were often considered by the public as things that girls should be good at. Under such a social and cultural background, girls believed that they should have good reading and summary ability, while boys who even knew how to apply strategies to summarize an article would underestimate their ability. As a result, girls’ metacognitive summarizing strategies cannot effectively predict their reading self-efficacy, while boys’ metacognitive summarizing strategies can negatively predict their reading self-efficacy. In addition, the negative influence of stereotypes also affected the relationship between metacognitive understanding and remembering strategies and reading self-efficacy of boys and girls. Understanding and memory were classified as low-order thinking ability in Bloom’s educational goal classification theory. The concept of “low-order” was often despised and the educational environment such as schools often did not support “rote memorization” (Fata-Hartley 2011). Therefore, students tended to underestimate the role of metacognitive understanding and remembering strategies, leading to the negative prediction of reading self-efficacy in metacognitive understanding and remembering strategies.

### 6.4. Limitations and Implications for Future Research

There are several limitations to the study. First of all, the data in the PISA 2018 dataset were used for analysis in this study, and the survey adopted a cross-sectional design, so the study could not confirm causality. In the future, longitudinal or experimental research design could be used to conduct research, which would help to strengthen or develop the thesis of this study. Secondly, this study only used the data in the database for quantitative analysis, and the database did not provide relevant qualitative cases. Therefore, qualitative data could be supplemented or mixed research could be carried out in the future to reveal the relationship among metacognitive reading strategies, reading self-efficacy, reading literacy and scientific literacy. Third, this study only focused on metacognitive reading strategies from three sub-dimensions provided by the PISA 2018. Text processing usually involves more than understanding and memory, summarizing, and information credibility assessment. In the future, more metacognitive strategies in text-processing tasks could be developed, as a more comprehensive assessment of a wide range of metacognitive strategies plays an important role in scientific literacy. Last but not least, when using the PISA or large sample database for SEM analysis, it is necessary to carefully interpret significance according to *p*-value, especially when the path has a small beta coefficient, to avoid disseminating irrelevant information to the scientific discourse community.

### 6.5. Practical Implications for Science Education

The study has implications for promoting scientific literacy among students. First of all, teachers need to pay attention to the cultivation of metacognitive reading strategies and reading literacy. Science teachers can teach students strategies for reading scientific articles in the classroom and give them time for practice and reflection. They can help students learn how to sift through useful and reliable information in scientific articles, understand key scientific concepts, construct networks of relationships between concepts and learn to summarize scientific articles. Although the emphasis of science teaching is to enable students to solve scientific problems, the solution of scientific problems is still inseparable from the text-processing ability of students. Do not let the lack of reading literacy become a stumbling block to the cultivation of students’ scientific literacy.

Moreover, teachers and parents should reduce the negative influence of social and cultural stereotypes on students’ scientific literacy. Reading self-efficacy is an important factor in the influence path of students’ scientific literacy. Social and cultural stereotypes have varying degrees of influence on students’ sense of reading self-efficacy, making girls think that they should have good reading ability, while boys tend to underestimate their reading ability. Therefore, it is suggested that teachers and parents work together to address the impact of gender differences on students’ scientific literacy and reduce the stereotypical attitudes and behaviors caused by supporting gender differences.

## 7. Conclusions

This study showed that the three metacognitive reading strategies all played a role in promoting scientific literacy, and the metacognitive assessing credibility strategies had the most positive impact on scientific literacy. Among them, reading literacy played an important role in the influence of three metacognitive reading strategies on scientific literacy, which indicated that teachers need to attach importance to the teaching of reading strategies in science teaching. Moreover, there were gender differences in the effects of the three metacognitive reading strategies on scientific literacy. The reading self-efficacy of boys and girls had different degrees of negative effects on the effects of metacognitive summarizing strategies on scientific literacy. This difference may be caused by stereotypes in social culture. Therefore, we suggest that teachers and parents need to work together to reduce the negative effects of stereotypes on the development of students’ scientific literacy.

## Figures and Tables

**Table 1:** Descriptive statistics and correlation analysis.

	Mean				1	2	3	4	5
	Total	Boys	Girls						
1. UNDREM	0.210	0.089	0.341	0.994	1				
2. METASUM	−0.108	−0.228	0.022	0.962	0.483 *				
3. METASPAM	0.098	0.006	0.198	0.963	0.345 *	0.377 *			
4. SELEFCY	0.083	0.073	0.094	0.861	−0.024 *	−0.009	0.061 *		
5. READ	563.153	555.898	571.032	85.187	0.362 *	0.400 *	0.502 *	0.213 *	
6. SCI	595.977	600.120	591.478	79.645	0.334 *	0.378 *	0.478 *	0.187 *	0.938 *

* *p* < 0.05.

**Table 2:** The effects of each action path.

Path	Effect	SEx	*p*	95% Confidence Interval	
				Lower	Upper
UNDREM→SCI	−0.01	0.004	0.011	−0.018	−0.002
UNDREM→READ→SCI	0.137	0.009	0.001	0.121	0.154
UNDREM→SELEFCY→READ→SCI	−0.008	0.002	0.001	−0.012	−0.004
Total Mediation Effect (UNDREM→SCI)	0.129	0.009	0.001	0.112	0.146
Total Effect (UNDREM→SCI)	0.120	0.009	0.001	0.101	0.138
METASUM→SCI	0.004	0.004	0.316	−0.004	0.012
METASUM→READ→SCI	0.18	0.009	0.001	0.163	0.197
METASUM→SELEFCY→READ→SCI	−0.004	0.002	0.084	−0.007	0
Total Mediation Effect (METASUM→SCI)	0.177	0.009	0.001	0.16	0.194
Total Effect (METASUM→SCI)	0.181	0.01	0.001	0.163	0.2
METASPAM→SCI	0.011	0.004	0.006	0.004	0.019
METASPAM→READ→SCI	0.342	0.007	0.001	0.327	0.356
METASPAM→SELEFCY→READ→SCI	0.015	0.002	0.001	0.011	0.019
Total Mediation Effect (METASPAM→SCI)	0.357	0.008	0.001	0.342	0.371
Total Effect (METASPAM→SCI)	0.368	0.008	0.001	0.352	0.384

**Table 3:** The fitting index of the path structural equation model.

Type	χ^2^/*df*	*p*	RMSEA	SRMR	GFI	AGFI	NFI	IFI	CFI	TLI
Total	4.408	0.000	0.017	0.013	0.998	0.995	0.998	0.999	0.999	0.997
Boys	3.852	0.05	0.022	0.0017	1.000	0.995	1.000	1.000	1.000	0.998
Girls	14.929	0.000	0.05	0.0036	0.999	0.981	0.999	0.999	0.999	0.987

## Data Availability

The data in PISA 2018 can be downloaded at: https://www.oecd.org/pisa/data/2018database/.

## Supplementary Materials

The following supporting information can be downloaded at: https://www.mdpi.com/article/10.3390/jintelligence11050078/s1, File S1: Metacognitive Reading Strategies Measurement Tool in PISA 2018.
