# Supplementary material for: Research on the Influence Path of Metacognitive Reading Strategies on Scientific Literacy
Source: J Intell. 2023 Apr 23;11(5):78. doi: 10.3390/jintelligence11050078 (PMC10218841; doi:10.3390/jintelligence11050078)
Supplement: Supplementary file 1 [file jintelligence-11-00078-s001.zip › jintelligence-2219112-supplementary.pdf]

Metacognitive Reading Strategies Measurement Tool in PISA2018

There are several strategies to read texts. Some of them are more useful or appropriate than others, depending on the kind of reading task. The next questions present three different reading tasks, followed by a list of "strategies". We want to know your opinion about the usefulness of these strategies for the different reading tasks.

Think about the usefulness of each of the strategies in relation to the given reading task only. Some strategies may be useful for one reading task but not for another.

Give a score between 1 and 6 to every strategy. A score of 1 means you think it is not a useful strategy at all for this reading task. A score of 6 means you think it is a very useful strategy for this reading task.

You can use the same score more than once if you think two or more strategies are similarly useful, but please select only one response in each row.

Metacognitive understanding and remembering strategies

Reading task: You have to understand and remember the information in a text.

How do you rate the usefulness of the following strategies for understanding and memorising the text?

No.	Strategies	Score (1-6): from not useful at all to very useful
1	I concentrate on the parts of the text that are easy to understand.	
2	I quickly read through the text twice.	
3	After reading the text, I discuss its content with other people.	
4	I underline important parts of the text.	
5	I summarise the text in my own words.	
6	I read the text aloud to another person.	

Metacognitive summarizing strategies

Reading task: You have just read a long and rather difficult two-page text about fluctuations in the water level of a lake in Africa. You have to write a summary.

How do you rate the usefulness of the following strategies for writing a summary of this two-page text?

No.	Strategies	Score (1-6): from not useful at all to very useful
1	I write a summary. Then I check that each paragraph is covered in the summary, because the content of each paragraph should be included.	
2	I try to copy out accurately as many sentences as possible.	
3	Before writing the summary, I read the text as many times as possible.	
4	I underline important parts of the text.	
5	I carefully check whether the most important facts in the text are represented in the summary.	
6	I read through the text, underlining the most important sentences. Then I write them in my own words as a summary.	

Metacognitive assessing credibility strategies

Reading Task: You have received a message in your inbox from a well-known mobile phone operator telling you that you are one of the winners of a smartphone. The sender asks you to click on the link to fill out a form with your data so they can send you the smartphone.

In your opinion, how appropriate are the following strategies in reaction to this email?

No.	Strategies	Score (1-6): from not useful at all to very useful
1	Answer the email and ask for more information	

	about the smartphone	
2	Check the sender’s email address	
3	Click on the link to fill out the form as soon as possible	
4	Delete the email without clicking on the link	
5	Check the website of the mobile phone operator to see whether the smartphone offer is mentioned	
